# Survival and Prognostic Factors for AIDS and Non-AIDS Patients with Non-Hodgkin's Lymphoma in Bahia, Brazil: A Retrospective Cohort Study

**DOI:** 10.1155/2013/904201

**Published:** 2013-10-29

**Authors:** Estela Luz, Marinho Marques, Ivana Luz, Cristiani Stelitano, Eduardo Netto, Iguaracyra Araújo, Carlos Brites

**Affiliations:** ^1^Hospital Universitário Professor Edgard Santos, Universidade Federal da Bahia, Rua Augusto Viana s/n, 40110-060 Salvador, BA, Brazil; ^2^Serviço de Hematologia, Núcleo de Oncologia da Bahia, Avenida Adhemar de Barros 123, Ondina, 40170-110 Salvador, BA, Brazil

## Abstract

Despite the benefits of HAART, HIV-infected patients are increasingly affected by different malignancies. We compared a 5-year-period survival time and prognostic factors for HIV-1-infected individuals diagnosed with non-Hodgkin lymphomas (NHL) in a nested case-control study, with non-HIV-infected individuals in Salvador, Brazil. Survival time and prognostic factors were compared to HIV-negative patients. 31 cases (versus 63 controls) had a significantly more advanced NHL at diagnosis and lower mean CD4 count (26 cells/mm^3^) than controls. Mean overall survival (OS) was 35.8 versus 75.4 months, for cases and controls, respectively (*P* < 0.001), while mean event-free survival time (EFS) was 34.5 months for cases, versus 68.8 for controls (*P* = 0.002). Higher IPI, increased LDH levels, bone marrow infiltration, lower absolute lymphocyte counts (<1,000 cells/mm^3^), and type B symptoms were associated with a shorter survival time for cases. Although patients without poorer prognostic factors at baseline had an OS comparable to controls, the mean CD4 cell count for cases was similar for patients with favorable and nonfavorable response to therapy. Our findings suggest that HIV-1 infection is significantly associated with a shorter survival time for patients with NHL, independently of other predictive factors and of disease stage.

## 1. Introduction

The main characteristic of AIDS is a severe immunodeficiency caused by the progressive depletion of CD4+ T-cells and consequent immune impairment [1]. These facts ultimately lead to an increasing risk of developing opportunistic infections and malignancies [2, 3]. Non-Hodgkin lymphoma (NHL) is an AIDS-defining condition, and its frequency seems to be decreasing after the highly active antiretroviral therapy (HAART) became the standard of care for the management of AIDS patients [4, 5]. HAART has promoted a great increase in the lifespan of HIV-infected patients and decreased the morbidity and mortality caused by AIDS [6]. However, the available evidence suggests that around half of the patients treated are unable to reach a normal CD4+ cell count, even after years of persistent viral suppression. One of the reasons for such findings is the persistent immune activation, but its causative mechanisms are still a matter of debate [7]. 

These facts may be responsible for the persistence of a higher risk for the development of other malignancies, a trend recently detected in treated AIDS patients, as a likely consequence of incomplete immune restoration. While there is a clear trend toward reducing AIDS-defining events (which include Kaposi's sarcoma and non-Hodgkin lymphomas) after HAART introduction, several published works have shown an increase in the frequency of other malignancies among AIDS patients under suppressive therapy, including some cancers not previously associated with HIV infection, such as Hodgkin's lymphoma (HL). Although the majority of the neoplasms identified are anal and lung cancer, an increment in the frequency of diagnosis of Hodgkin's disease among HIV-infected patients has also been observed [4, 8–11].

In Brazil, since the beginning of the AIDS epidemic, the Federal Government has provided universal access to antiretroviral drugs, for all patients in need of treatment. This strategy was shown to be very efficacious in promoting an increase in the survival time for AIDS patients, as well as in stabilizing the number of cases over time. Currently, the life expectation of HIV-infected individuals is approaching found in the general population. However, the available information suggests that there is an increase in the frequency of malignancies among Brazilian HIV-infected patients, as observed in other countries [12, 13]. Unfortunately, there is scarce information on the frequency of each type of cancer and on survival time for HIV-related malignancies. In this paper, we present an evaluation of survival time and prognostic markers for AIDS patients presenting with NHL.

## 2. Patients and Methods

### 2.1. Study Design

This was a retrospective, nested, case-control study (1 exposed for 2 nonexposed) within a cohort of HIV patients receiving follow-up at the AIDS clinics of Complexo Hospitalar Universitário Professor Edgard Santos (HUPES), the Federal University of Bahia (UFBA) teaching hospital. We also reviewed the medical records of oncology clinics in Salvador, Bahia, Brazil, to include HIV-negative controls.

### 2.2. Study Population

#### 2.2.1. Case Definition

Patients were with confirmed diagnosis of non-Hodgkin's lymphomas (NHL) between 2000 and 2006, who had available information on follow-up until December 2009, older than 18 years, with a confirmed diagnosis of HIV infection. 

#### 2.2.2. Control Definition

Patients met all case criteria, but a negative serology for HIV infection. Cases and controls were matched by gender, age (±5 years), and same year of diagnosis.

All individual medical charts were reviewed for data collection on demographics, disease staging (Ann Arbor classification system [14]), ECOG (Eastern Cooperative Oncology Group [15]), response to therapy, type B symptoms (fever, weight loss, and night sweats), survival time, laboratory and imaging evaluations, bulky disease (mass ≥ 10 cm), and bone marrow infiltration evidence. For the International Prognosis Index (IPI) [16] five variables were used (age, disease staging, number of extra nodal sites, ECOG and LDH levels).

### 2.3. Statistical Analysis

Demographics were compared using chi-square test (comparison of proportions) or student's *t*-test (means). Kaplan-Mayer survival analysis was performed to compare total survival (up to death) and event free survival (EFS), considering the period between the date of diagnosis and the date of death or relapse, end of or losing follow-up. Data was collected until December 2009. Event-free survival (EFS) was calculated from date of diagnosis up to the date of disease recurrence or end of follow-up. All statistical analysis was performed by using the software package SPSS for Windows, version 17.0.

The study was approved by the Ethics Committee of the Maternidade Climério de Oliveira-Universidade Federal da Bahia (approval no.: 0648/2008). 

## 3. Results

Thirty-one AIDS patients with a diagnosis of NHL between 2000 and 2006 were included in the study, as well as 62 HIV-negative unexposed controls, which had the same diagnosis, matched by age, gender, and year of diagnosis of NHL.

Table 1 shows the baseline characteristics of both groups. Of note, AIDS patients had at diagnosis a significantly higher frequency (42.1%) of ECOG ≥ 2 than negative controls (10.7%, *P* = 0.005). In addition, we detected a higher proportion of exposed patients (84.2%) with an IPI ≥ 2, compared to unexposed controls (51.8%, *P* = 0.01). Advanced disease stage (stage III/IV) was also a more frequent finding among exposed patients (77.8%) than among unexposed controls (55.7%) although it was only marginally significant (*P* = 0.05). The groups were comparable regarding the other demographic and clinical characteristics.

Most of the exposed cases (58.1%) had received an HIV diagnosis before the detection of NHL, and the mean time between HIV diagnosis and NHL detection was 32 months (range 4–144 months). In 13 patients (41.9%) the diagnosis was performed simultaneously, during clinical investigation due to a delay in request HIV serology. The majority (28/31 patients, 90.3%) of AIDS patients had a diffuse large B-cell lymphoma (three had the immunoblastic variant and 4 the plasmablastic one). The remaining patients did not have information about variant morphological of the lymphoma available in their medical records. Three patients had Burkitt's lymphoma (two as the classic form and the third as the atypical one). All controls were classified as diffuse large B-cell lymphoma.

The HIV staging at diagnosis of lymphoma showed that 14 (45.2%) had a CD4+ cell count below 200 cells/mm^3^. The mean CD4+ cell count was 211 cells/mm^3^ (range 4–683), while the mean plasma HIV-1 RNA viral load was 96,000 copies/mm^3^ (range < 40–350,000 copies/mm^3^). Mean absolute lymphocyte count was similar for cases (mean 1499 cells/mm^3^, range 260–4000) and controls (mean 1536 cells/mm^3^, range 110–3906, *P* = 0.77). Lactate dehydrogenase (LDH) levels were also similar for both groups (808 mg/dL, range 144–3520 for cases, versus 132 mg/dL, range 117–7791 for controls, *P* = 0.81).

All patients were treated with anthracycline-based chemotherapy regimens (CHOP or similar). Among those exposed to HIV infection, 5 patients died before starting chemotherapy. Five patients were also submitted to radiotherapy but the remaining 21 only received chemotherapy. Twelve patients (38.7%) were undergoing HAART at the moment of diagnosis, and four died before starting antiretroviral therapy. Among the controls, only one was not treated. The majority (38 patients) were submitted to chemotherapy alone, while the remaining 23 received chemotherapy plus radiotherapy. 

The mean CD4 count (*N* = 26) was 211 cells/mm^3^ (range 4–683), while HIV-1 RNA plasma viral load (*N* = 23) varied from <40 to 350,000 copies/mm^3^. The mean absolute lymphocyte count was similar for HIV positive (1499 cells/mm^3^, range 260–4000 cells/mm^3^) and negative patients (1536 cells/mm^3^, range 110−3906, *P* = 0.77). 

### 3.1. Response to Treatment

#### 3.1.1. HIV-Positive Group

Five patients (19%) died before starting treatment. Twenty-two patients were treated with chemotherapy, and four cases received chemotherapy plus radiotherapy. A complete response to therapy was obtained by nine patients (34.6%), while 16 individuals (61.5%) presented with disease recurrence and eventually died. Median CD4 count was similar for patients successfully treated (152 ± 85 cells/mm^3^) and for those who failed therapy (127.5 ± 35 cells/mm^3^, *P* > 0.05).

#### 3.1.2. HIV-Negative Group

Only 1 out of 62 patients was not treated (early death). Twenty-three received chemotherapy plus radiotherapy, while the remaining 38 were treated with chemotherapy alone. Complete remission was achieved in 41 patients (67.2%) while 19 (31%) presented disease progression and died. Among the responders, 11 presented disease recurrence (7 died, 2 were lost to follow-up, and 2 presented subsequent sustained clinical remission).

**Figure 1:**
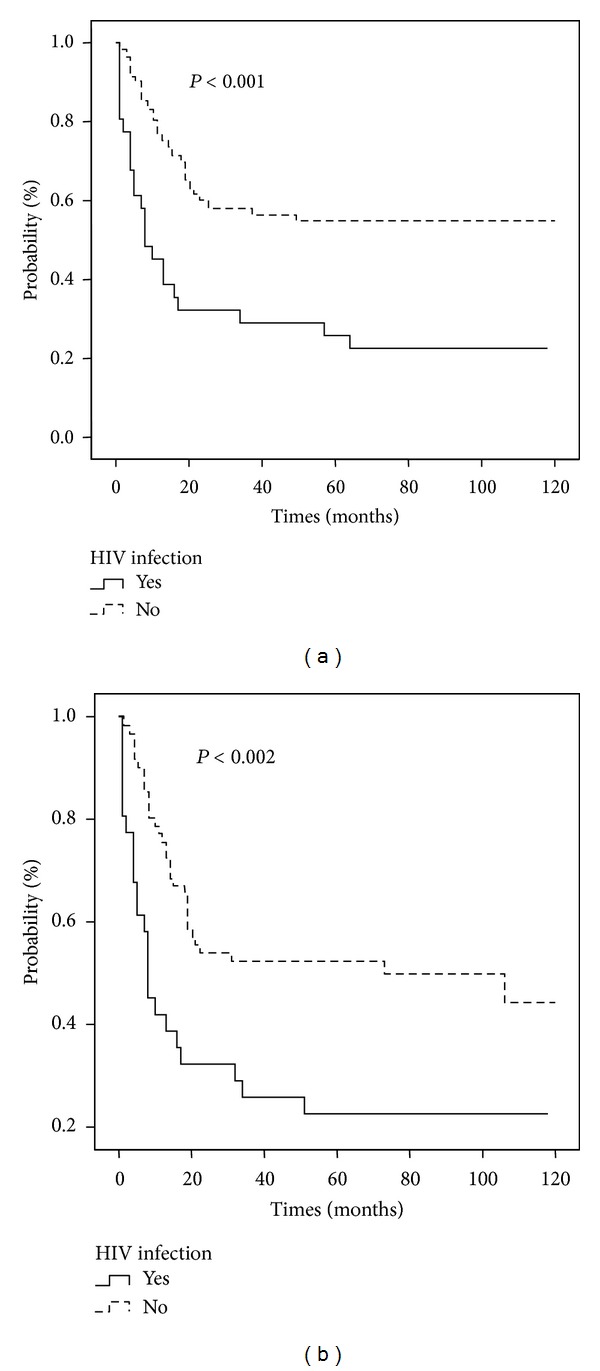
Overall survival (a) and event-free survival (b) for NHL patients according to HIV-1 infection status.

Overall survival time was significantly different for exposed and nonexposed controls, as shown in Figure 1. Exposed patients had a global mean survival of 35.8 months (range 1–120 months), while for controls it was equal to 72.4 months (range 1–120, *P* < 0.001). A similar pattern was observed when we evaluated the event-free survival time (EFST), with cases showing a shorter (34.5 months) mean EFST than controls (66.8 months, *P* = 0.002). 

The main predictors of shorter survival for AIDS patients were IPI ≥ 2, presence of bulky disease, increased DHL levels, bone marrow infiltration, absolute lymphocyte count < 1.000/mm³, and presence of type B symptoms.  Table 2 summarizes these findings. In addition, HIV-positive patients had a significantly higher rate of IPI ≥ (*P* = 0.02), higher LDH levels (*P* < 0.001), lower absolute lymphocyte count (*P* = 0.01), and a higher frequency of type B symptoms (*P* = 0.007) than seronegative individuals. They also had higher and earlier mortality than the control group, even when it was adjusted for the presence of these parameters (Table 3).

A similar picture was observed when we compared predictive factors for EFS; AIDS patients presented a significantly higher frequency of bulky disease, bone marrow infiltration, and type B symptoms, as well as a lower absolute lymphocyte count and higher LDH levels than controls. Tables 2 and 3 summarize these findings. The overall survival of AIDS patients without poor prognostic findings was similar to that found for HIV-negative individuals with those characteristics.

## 4. Discussion

The present study demonstrates that overall survival time and EFS for patients diagnosed with NHL are significantly reduced by HIV infection. While HIV-negative patients had a global survival and event-free survival of 72.4 months and 58.9 months, respectively, these time intervals were reduced to 35.8 and 34.5 months, respectively, when patients were HIV positive. The main predictors of a shorter survival for HIV-positive patients were IPI ≥ 2, bulky disease, increased LDH levels, low absolute lymphocyte count (<1,000 cells/mm^3^), and presence of B symptoms.

The incidence of NHL in HIV patients varies from 1–3% and was higher in the pre-HAART era [17, 18]. During the study period, 2,274 AIDS cases were reported to health authorities in Salvador, and 31 patients (1.4%) with NHL were identified. This incidence rate is similar to that reported in other studies, but in our case it can underestimate the real rate, since we were not able to cover all the oncology services in town, and, in some cases HIV screening was not performed. It is noteworthy that the diagnosis of NHL was reached in patients previously diagnosed with AIDS in almost 2/3 (58%) of them, and in 39%, it was the first manifestation of the disease. The mean time for NHL diagnosis after detection of HIV infection was 31 months but was as long as 166 months in one case. In addition, 45% of these patients had a CD4+ cell count lower than 200 cells/mm^3^. This confirms NHL as a late manifestation of AIDS, as well as the findings detected by other studies [7]. 

The vast majority (90.3%) of HIV-positive patients in our study had LDBCL, an AIDS-defining disease since 1993. This proportion was similar to that found by Pereira (80%), in São Paulo [19], and Barreto (61%) in Rio de Janeiro [20], both located in Brazil. In our study, all HIV-negative patients had a diagnosis of LDBCL, which allowed us a better comparison of survival time for groups with the same disease but with different immunological conditions. Although it is well known that other viral agents can play a role in lymphomagenesis, we could not evaluate this factor for our patients, due to the lack of serological information in a great number of patients. Of note, among the HIV-positive group we detected a 6.5% coinfection rate by hepatitis C virus (2 patients), and only one case (3.2%) coinfected by HTLV-1. For comparison purposes, Simcock et al. [21] found a 27.5% coinfection rate by HCV, among AIDS patients with NHL [21]. In addition, the coinfection rates we have already found in our overall population of HIV patients is much higher than this, with coinfection by HCV reaching 10–15% and by HTLV varying from 8% to 16% [22]. It is important to emphasize that these patients need a better evaluation, regarding the screening for potentially coinfecting agents, since the simultaneous infection by other viruses can have an impact on the clinical evolution and outcomes of these individuals.

The overall survival time and EFS were similar for patients without poor prognostic factors. However, when these findings were present, being HIV positive was a significant negative factor for survival. An IPI equal or higher than two was significantly associated with a shorter OS and EFS, for HIV-positive patients, as well as the presence of other poor prognostic factors, like those reflecting a greater tumor load (bulky disease and elevated LDH), or advanced/disseminated disease (bone marrow involvement and presence of type B symptoms). These findings were similar to those reported by Simcock et al. [21]. 

Another independent factor associated with a poorer prognosis was an absolute lymphocyte count below 1,000 cells/mm^3^. Patients with a higher absolute lymphocyte count had a similar OS and EFS, regardless of their HIV serostatus. However, for patients infected by HIV, a lymphocyte count below that threshold was significantly associated with a shorter survival. This fact suggests that immunosuppression is a risk factor not only for the development of neoplasms, but it is also an important negative prognostic factor. Our findings were similar to those described by Kim et al. in 2007 [23]. On the other hand, it seems that the CD4+ cell count was not associated with the response to treatment, since patients with complete response had similar baseline CD4+ cell count to those who presented with progressive disease even after chemotherapy. The potential mechanisms to explain the association between decreased absolute lymphocyte count and shorter survival time include (a) the factors released by the tumor cells, which could cause suppression of dendritic cells and T cells [24]; (b) the effect of chemotherapy, which can transiently intensify the degree of immunosuppression, increasing the risk of potentially lethal infections; and (c) a combination of these factors [25, 26].

Taken together, these findings suggest that the infection by HIV-1 is significantly associated with a shorter survival time for patients presenting with NHL. Our results confirm HIV-1 as a negative prognostic factor for such patients, regardless of other predictive factors and of the disease stage for both NHL and HIV infection. 

## Figures and Tables

**Table 1:** Characteristics of patients with NHL according to HIV status, at the moment of diagnosis, in Salvador-Bahia.

Variable	HIV positive		HIV negative		*P* value
	n = 31	%	n = 62	%	
Age in years (means ± SDP)	44.1 ± 8.6		44.1 ± 10.6		0.99
Gender					
Male	19	61.3	38	61.3	1.00
Female	12	38.7	24	38.7	
Disease stage					
I/II	6	22.2	27	44.3	**0.05**
III/IV	21	77.8	34	55.7	
Bulky disease					
Yes	8	29.6	20	32.8	0.81
No	19	70.4	41	67.2	
Number of extranodal sites					
0 and 1	17	58.6	51	82.2	0.10
2 and 3	12	41.3	11	17.7	
B symptoms					
Yes	24	85.7	42	68.9	0.12
No	4	14.3	19	31.1	
ECOG					
<2	11	57.9	50	89.3	**0.005**
≥2	8	42.1	6	10.7	
LDH					
Normal	5	21.7	23	41.1	0.10
Elevated	18	78.3	33	58.9	
IPI					
<2	3	15.8	27	48.2	**0.01**
≥2	16	84.2	29	51.8	
Bone marrow infiltration					
Yes	5	21.7	9	17.6	0.75
No	18	78.3	42	82.4	

**Table 2:** Predictive factors of overall survival for patients with NHL, according to HIV serostatus.

Characteristics	HIV positive			HIV negative			
	N	Mean (months)	IC	*N*	Mean (months)	IC	P value
ECOG							
0-1	11	57	24.4–88.7	50	77	62.3–90.8	0.187
2-3	8	5	1.7–7.5	6	28	1.9–54.5	0.175
IPI							
0-1	3	80	22.4–137.6	27	94	77.8–110.9	0.680
2-3	16	25	5.0–45.5	29	51	33.2–69.2	**0.017**
Bulky disease							
Yes	8	14	0–28.6	20	53	29.7–76.0	**0.004**
No	19	52	28.0–75.0	41	81	65.7–97.1	**0.036**
LDH							
Normal	5	74	28.5–119.1	23	83	69.4–108.4	0.605
Elevated	18	19	3.3–34.3	33	62	45.1–82.1	**<0.001**
Bone marrow infiltration							
Yes	5	5	0.3–9.7	9	36	14.5–57.8	**0.001**
No	18	54	29.5–78.3	42	84	68.7–99.5	**0.026**
Disease stage							
1 or 2	6	69	29.0–108.6	27	97	80.2–113.2	0.184
3 or 4	21	31	12.9–49.0	34	51	33.7–67.4	0.067
Absolute lymphocyte count (>1,000/mm³)							
Yes	16	49	22.5–75.2	40	73	57.4–89.6	0.064
No	6	6	2.4–10.2	17	53	28.8–78.6	**0.011**
B symptoms							
Yes	24	34	15.5–51.5	42	64	47.5–79.9	**0.007**
No	4	70	21.1–117.9	19	91	69.2–112.8	0.411

**Table 3:** Predictive factors for EFS for patients with NHL, according to HIV serostatus.

Factors	HIV positive			HIV negative			
	*N*	Mean (months)	IC	*N*	Mean (months)	IC	*P* value
ECOG							
0-1	11	57	24.4–88.7	50	75	60.5–89.8	0.312
2-3	8	4	2.0–6.0	6	28	1.9–54.5	0.084
IPI							
0-1	3	80	22.4–137.6	27	93	75.4–110.2	0.952
2-3	16	25	4.6–45.2	29	50	31.9–68.3	0.024
Bulky disease							
Yes	8	10	2.5–17.5	20	52	28.8–75.5	0.007
No	19	51	27.3–74.6	41	78	62.0–93.0	0.082
LDH							
Normal	5	74	28.5–119.1	23	85	65.7–104.2	0.958
Elevated	18	19	3.0–34.0	33	62	43.3–81.0	<0.001
Bone marrow infiltration							
Yes	5	4	0.8–7.2	9	33	14.5–57.8	0.002
No	18	52	27.6–76.6	42	83	67.4–98.8	0.102
Disease stage							
1-2	6	69	29.0–108.6	27	96	79.4–113.0	0.413
3-4	21	29	11.2–46.6	34	49	31.8–66.1	0.092
Absolute lymphocyte count (>1,000/mm³)							
Yes	16	49	22.5–75.2	40	72	55.4–88.4	0.134
No	6	6	2.4–8.6	17	53	28.2–78.4	0.012
B symptoms							
Yes	24	32	13.9–49.6	42	62	45.6–78.7	0.012
No	4	70	21.1–117.9	19	88	66.6–109.0	0.708
